# Aliskiren-attenuated myocardium apoptosis *via* regulation of autophagy and connexin-43 in aged spontaneously hypertensive rats

**DOI:** 10.1111/jcmm.12273

**Published:** 2014-04-06

**Authors:** Wenbin Zhang, Gang Zhao, Xiaona Hu, Min Wang, Hua Li, Yong Ye, Qijun Du, Jin Yao, Zhijun Bao, Wei Hong, Guosheng Fu, Junbo Ge, Zhaohui Qiu

**Affiliations:** a Department of Cardiology, Biomedical Research (Therapy) Center, Sir Run Run Shaw Hospital, College of Medicine, Zhejiang UniversityHangzhou, Zhejiang, China; b Shanghai Institute of Cardiovascular Diseases of Zhongshan Hospital, Fudan UniversityShanghai, China; c Gastroenterology Department, Huadong Hospital, Fudan UniversityShanghai, China; d Shanghai Key Laboratory of Clinical Geriatric MedicineShanghai, China; e Institute of Biomedical Science, Fudan UniversityShanghai, China; f Cardiovascular Department, Huadong Hospital, Fudan UniversityShanghai, China; g Geriatrics Department, Huadong Hospital, Fudan UniversityShanghai, China

**Keywords:** connexin-43, aged spontaneously hypertensive rats, apoptosis, autophagy, Aliskiren, Survivin, AKT, Caspase3

## Abstract

There are controversies about the mechanism of myocardium apoptosis in hypertensive heart disease. The aim of this study was to investigate the relationship among autophagy, Cx43 and apoptosis in aged spontaneously hypertensive rats (SHRs) and establish whether Aliskiren is effective or not for the treatment of myocardium apoptosis. Twenty-one SHRs aged 52 weeks were randomly divided into three groups, the first two receiving Aliskiren at a dose of 10 and 25 mg/kg/day respectively; the third, placebo for comparison with seven Wistar-Kyoto (WKY) as controls. After a 2-month treatment, systolic blood pressure (SBP), heart to bw ratios (HW/BW%) and angiotensin II (AngII) concentration were significantly enhanced in SHRs respectively. Apoptotic cardiomyocytes detected with TUNEL and immunofluorescent labelling for active caspase-3 increased nearly fourfolds in SHRs, with a decline in the expression of survivin and AKT activation, and an increase in caspase-3 activation and the ratio of Bax/Bcl-2. Myocardium autophagy, detected with immunofluorescent labelling for LC3-II, increased nearly threefolds in SHRs, with the up-regulation of Atg5, Atg16L1, Beclin-1 and LC3-II. The expression of Cx43 plaque was found to be down-regulated in SHRs. Aliskiren significantly reduced SBP, HW/BW%, AngII concentration and the expression of AT_1_R. Thus, Aliskiren protects myocardium against apoptosis by decreasing autophagy, up-regulating Cx43. These effects showed a dose-dependent tendency, but no significance. In conclusion, the myocardium apoptosis developed during the hypertensive end-stage of SHRs could be ameliorated by Aliskiren *via* the regulation of myocardium autophagy and maladaptive remodelling of Cx43.

## Introduction

Hypertension causing harmful complications in cardiovascular system has been identified as major risk factor for cardiac mortality. Although many antihypertensive medications have been developed, ventricular hypertrophy and its subsequent congestive heart failure are still common occurrences in most hypertensive patients. Our previous study [1] and some other studies [2–5] demonstrated that apoptosis and autophagy, associated with cardiac hypertrophy and heart failure, might be a possible mechanism involved in the transition from stable compensatory heart failure to decompensation during the end-stage of hypertensive heart disease.

Connexin-43 (Cx43) is an important communication channel with which cardiomyocytes connect to each other electrically and metabolically [6–8]. It was also reported [9,10] that mitochondrial Cx43 plays an important role in apoptosis. The abundance and distribution of connexin proteins are altered under a variety of pathological conditions such as hypertrophy, heart failure and ischaemia [11]. Furthermore, previous studies associated the alteration of Cx43 with the apoptosis of myocardium in diabetic rats, which could be reversed by losartan and simvastatin [12]. In addition, autophagy has been reported [13,14] to play a key role in the degradation of connexin. However, the relation among myocardium apoptosis, autophagy and Cx43 in the end-stage of hypertensive disease is not yet known.

Aliskiren, a direct renin inhibitor newly developed, produced a marked therapeutic effect on hypertension [15,16] and hypertrophy [17]. However, no studies investigated whether Aliskiren could attenuate the cardiomyocyte apoptosis in the end-stage of hypertensive heart disease.

This study showed that the decreased expression and disorder of Cx43 could play a crucial role in this pathophysiological process, and Aliskiren could decrease the myocardium autophagy, ameliorate Cx43 remodelling and reduce myocardium apoptosis in aged SHRs.

## Material and methods

### Animals

Twenty-one male SHRs, aged 52 weeks (weighing ∼400 g) and seven age-matched normotensive male Wistar-Kyoto (WKY) were bred *ad libitum*. Spontaneously hypertensive rats were randomly divided into three groups: Aliskiren group at a low dose of 10 mg/kg/day (SHR+LA, *n* = 7; gift of Novartis, Basel, Switzerland); Aliskiren group at a high dose of 25 mg/kg/day (SHR+HA, *n* = 7); and SHR control group (SHR, *n* = 7). An additional group of WKY was treated as controls (WKY, *n* = 7). Aliskiren and vehicle were daily administered through an intra-gastric tube for 8 weeks.

All animal experimental procedures were approved by the Animal Care and Use Committee of Zhejiang University and performed in accordance with the Guide for the Care and Use of Laboratory Animals (NIH publication No. 85-23, National Academy Press, Washington, DC, USA, revised 1996). All surgery was performed under anaesthesia, with all efforts made to minimize suffering.

### Measurement of systolic blood pressure and heart to bw ratio

All the animals' systolic blood pressures (SBP) were measured at the beginning and then every 4 weeks under conscious conditions. The animals were trained to adapt themselves to the restraining cages and tail-cuff apparatus for the standard non-invasive tail-cuff method before the measurement [18]. Each test was repeated three times. The animals were killed by decapitation before their heart to bw ratios (HW/BW%) were calculated. Three rats in each group were used for western blot and qPCR, and the others were used for TUNEL, immunofluorescence and immunohistochemistry.

### Determination of plasma angiotensin II concentration

Blood was collected from aorta abdominalis at the end of the experiment; plasma angiotensin II concentrations determined by radioimmunoassay following SepPak extraction and high-performance liquid chromatography separation [19].

### Detecting apoptosis by TUNEL assay

*In situ* DNA fragments were detected using TUNEL method. Deparaffinized sections were pre-treated with protease K (20 μg/ml) for 20 min. at room temperature, followed by an incubation with 0.3% hydrogen peroxide in methanol for 5 min. at room temperature to quench endogenous peroxidase activity. The sections were treated with 1× TdT equilibration buffer for 30 min. and then incubated with terminal deoxynucleotidyl transferase for 90 min. at 37°C before visualized by streptavidin-biotin-peroxidase complex (TdT-FragELTM DNA fragmentation detection kit, Calbiochem, Merck, Darmstadt, German) and diaminobenzidine. From each rat were chosen three slides for TUNEL assays. The percentage of TUNEL-positive cells was calculated as follows: 400× (the number of TUNEL-positive cells counted/total number of nuclei counted and then normalized to that of WKY).

### Immunofluorescent labelling for active caspase-3 and LC3-II

The apoptosis and autophagy of cardiomyocytes were evaluated by Immunofluorescence staining of frozen sections with anti-active caspase-3 (rabbit polyclonal, 1:100; Abcam, Cambridge, MA, USA) or anti-LC3-II (1:200; Cell Signal Technology, Danvers, MA, USA) according to the manufacturer's direction. The sections derived from three to four slides of each group were examined and calculated.

### Immunohistochemical staining of Cx43

Each LV tissue was fixed in formalin for 48 hrs before embedded in paraffin and then sectioned into 5-μm-thick slides. The sections were deparaffinized, incubated with 0.3% hydrogen peroxide for 10 min. at room temperature to quench endogenous peroxidase activity, followed by an incubation of anti-Cx43 polyclonal antibody (1:200; Cell Signal Technology) at 4°C overnight. Washed three times with PBS for 10 min. each, the sections were incubated with a secondary goat anti-rabbit antibody labelled with horseradish peroxidase (HRP) for 30 min. at 37°C before visualized with diaminobenzidine under Leica DM-RE microscope (Brunswick, Germany).

### Quantitative real-time reverse transcriptional PCR determining the gene expression of Cx43, Atg5 and Atg16L1

RNA was isolated using TRIzol reagent (Invitrogen, Carlsbad, CA, USA) and the concentrations were determined by NanoDrop instrument (NanoDrop Technologies, Wilmington, DE, USA). SYBR RT-PCR kit (Takara, Dalian, China) was used for quantitative real-time PCR analyses, monitored by ABI PRISM 7900 System (Applied Biosystems, Carlsbad, CA, USA). The primers for Cx43 and GAPDH were designed by Takara:

For Cx43 gene, forward, 5′-AGGTCTGAGAGCCTGAACTCTCATT-3′ and reverse, 5′-GGCACTCCAGTCACCCATGT-3′.

For Atg5 gene, forward, 5′-TTTGACGCTGGTAACTGACAAAGTG-3′ and reverse 5′-CAAGGCAGAGCTGAGCTTGATG-3′.

For Atg16L1 gene, forward, 5′-GCTCCCGTGATGACCTGCTAA-3′ and reverse 5′-CTGAGCCTGCTGCCACGTAA-3′.

For GAPDH gene, forward, 5′-GTGCAGTGCCAGCCTCGTC-3′ and reverse, 5′-GGCAGCACCAGTGGATGCAG-3′.

The relative expression level of the gene was normalized to that of GAPDH by 2^−ΔΔCt^ cycle threshold method.

### Western blot analysis and quantification of Cx43, apoptotic regulatory proteins and autophagy proteins

The membrane and mitochondrial protein were extracted immediately after the rats' hearts were isolated, according to the operating instructions of Membrane Protein Extraction Kit (Boster, Wuhan, China) and Mitochondria Isolation Kit (Boster) respectively. The total proteins were extracted from the frozen ventricle tissues by RIPA. Total 40 μg samples were fractionated by SDS-PAGE and transferred to Immobilon-P membranes (Millipore, Billerica, MA, USA) as previously described [1], and then done with primary antibodies against Cx43, Bcl-2, Bcl-xL, Bax, AKT, phosphorylation of AKT (p-AKT), active Caspase-3, Beclin-1, LC3-II (1:1000, rabbit species, all from Cell Signal Technology) and survivin (1:1000, goat species, Santa Cruz Biotechnology, SantaCruz, CA, USA) at 4°C overnight. Rinsed three times in TBS, the filters blots were incubated with HRP-conjugated rabbit or goat second antibodies (1:5000; Kang Chen Biotechnology, Guangzhou, China) for 1 hr at room temperature. The bands were visualized by an ECL Western-blotting Detection Reagents (Catalogue RPN2106; GE Healthcare, Fairfield, CT, USA) with LAS-3000 detect system. To quantify the immunobloted bands, optic density was analysed using ImagePro 5.0 (Media Cybernetics Inc., Silver Spring, MD, USA).

### Statistical analysis

The results were analysed using one-way anova followed by Fisher's LSD test for multiple comparisons. Values were presented as mean ± SEM. *P* < 0.05 was considered statistically significant. All data were analysed using SPSS 16.0 statistical package (SPSS Inc., Chicago, IL, USA).

## Results

### Aliskiren down-regulated systolic blood pressure and decreased heart to bw ratio in aged SHRs

The average SBP was found to be higher in SHR controls than that in WKYs by 64% (*P* < 0.01), and decrease significantly in SHR+LA and SHR+HA by 14.1% and 17.6%, respectively, 4 weeks later (*P* < 0.01), and by 18.8% and 18.9%, respectively, 8 weeks later (*P* < 0.01). However, no significant difference was observed between SHR+LA and SHR+HA (*P* > 0.05; Table 1).

**Table 1:** Changes of SBP during the study

Group	SBP (mmHg)		
	
	Base line	At 4 weeks	At 8 weeks
WKY	126.00 ± 6.395	126.80 ± 6.224	119.40 ± 5.600
SHR	206.60 ± 5.316*	201.40 ± 3.881*	207.40 ± 2.159*
SHR+LA	203.80 ± 3.878*	175.00 ± 3.847#,*	165.40 ± 3.544#,*
SHR+HA	201.60 ± 6.185*	166.20 ± 3.023#,*	163.60 ± 2.482#,*

Values, mean ± SEM; *n* = 5–6; *P < 0.01 *versus* WKY controls; ^#^*P* < 0.01 *versus* SHR controls. SBP: systolic blood pressure; WKY: Wistar; SHR: spontaneously hypertensive rats; LA: low dose of Aliskiren; HA: high dose of Aliskiren.

HW/BW%, a parameter reflecting the size of the entire heart, was significantly enhanced by 94% in SHR controls when compared with WKYs, and decreased by 20% and 25% in SHR+LA and SHR+HA, respectively, at the end of the investigation (*P* < 0.01; Table 2).

**Table 2:** Changes of HW/BW% at the end of the study

Group	WKY	SHR	SHR+LA	SHR+HA
HW/BW%	0.21 ± 0.014	0.41 ± 0.029*	0.29 ± 0.033#,*	0.27 ± 0.017#,*

Values, mean ± SEM; *n* = 5–6; *P < 0.01 *versus* WKY controls; ^#^*P* < 0.01 *versus* SHR controls. HW/BW%: heart to bw ratio; WKY: Wistar; SHR: spontaneously hypertensive rats; LA: low dose of Alrrriskiren2; HA: high dose of Aliskiren.

### Aliskiren-attenuated myocardium apoptosis in aged SHRs

**Fig. 1:**
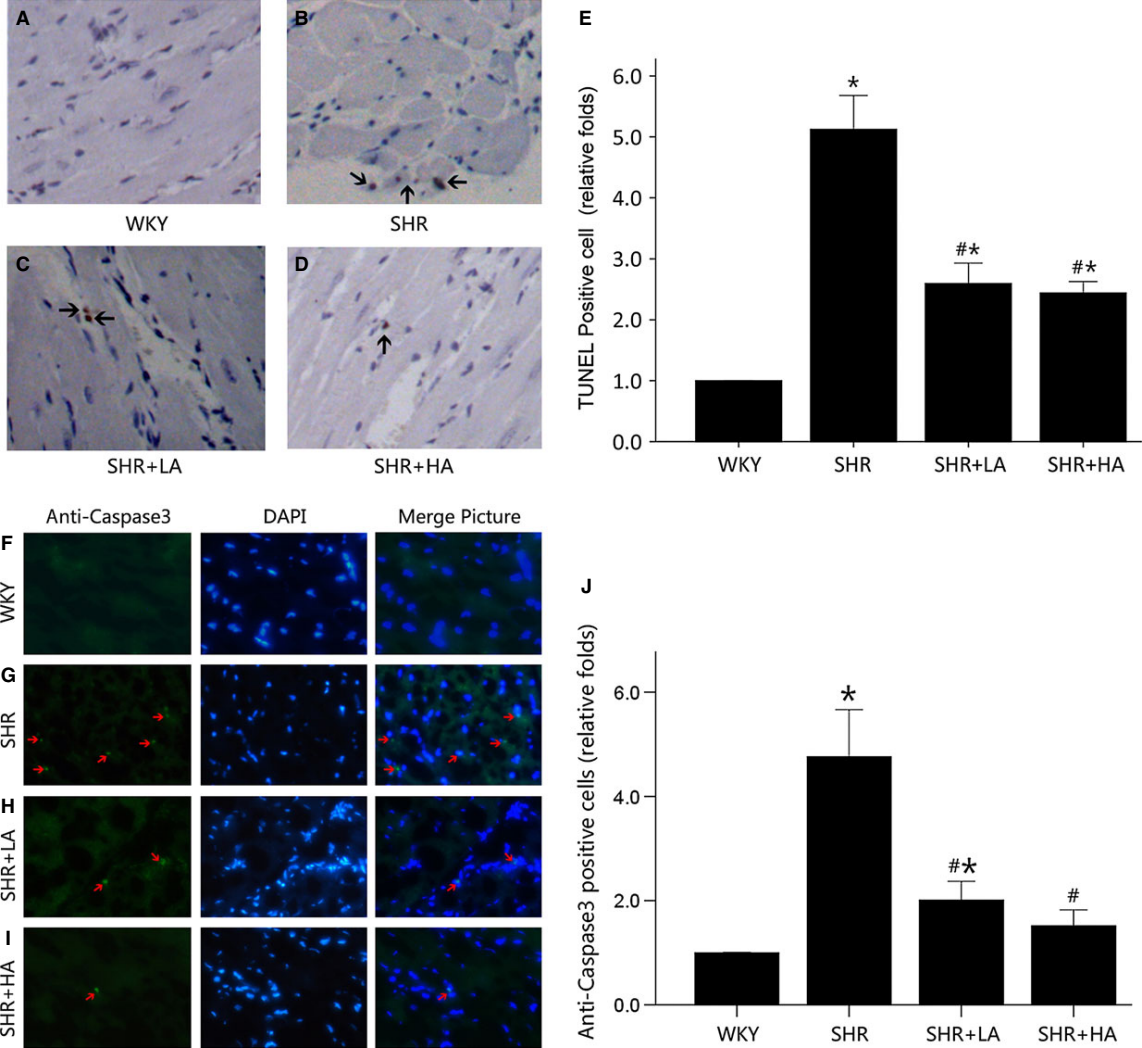
Cardiomyocyte apoptosis detected by TUNEL assay and Immunofluorescent labelling for active caspase-3. (**A**) No TUNEL-positive cardiomyocyte in the representative TUNEL staining of WKY controls; (**B**) multiple TUNEL-positive cardiomyocyte in the representative TUNEL staining of SHR controls compared with WKY controls (*P* < 0.01); (**C** and **D**) less TUNEL-positive cardiomyocyte in SHR+LA and SHR+HA (*P* < 0.01); (**E**) quantitative analysis of TUNEL-positive cardiomyocytes in the four groups by the ratio of TUNEL-positive cell number to the total and normalized to the WKY controls. (**F**) Few active caspase-3-positive cardiomyocytes in the representative immunofluorescent staining of WKY controls; (**G**) multiple active caspase-3-positive cardiomyocytes in the representative immunofluorescent staining of SHR controls compared with WKY controls (*P* < 0.05); (**H** and **I**) less active caspase-3-positive cardiomyocytes in SHR+LA and SHR+HA (*P* < 0.05); (**J**) quantitative analysis of active caspase-3-positive cardiomyocytes in the four groups by the ratio of active caspase-3-positive number to the total and normalized to the WKY controls. Values, mean ± SEM; *n* = 4; *P < 0.01 *versus* WKY controls; #*P* < 0.01 *versus* SHR controls; arrow indicating a TUNEL-positive cardiomyocytes or a caspase-3-positive cardiomyocyte; WKY: Wistar; SHR: spontaneously hypertensive rats; LA: low dose of Aliskiren; HA: high dose of Aliskiren.

TUNEL assay and immunofluorescence labelling for active caspase-3 showed that the number of apoptotic nuclei was nearly fourfolds in SHR controls when compared with WKYs (*P* < 0.01). SHR+LA displayed reductions in TUNEL-positive and active caspase-3-positive cell counts by 47.7% and 57.4%, respectively (*P* < 0.01), but with no significant difference between SHR+LA and SHR+HA (*P* > 0.05; Fig. 1).

**Fig. 2:**
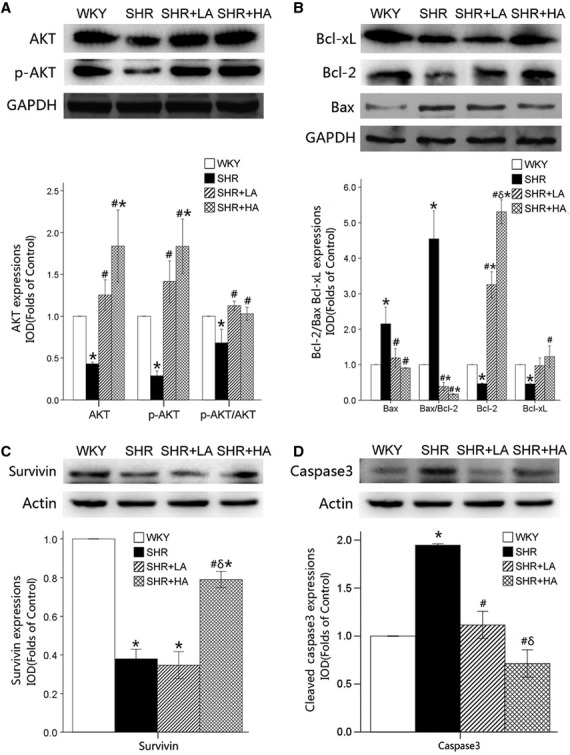
The expression of apoptotic regulatory protein revealed by western blot. (**A**) The expressions of p-AKT/AKT down-regulated significantly in SHR Controls (*P* < 0.01), and reversed in SHR+LA and SHR+HA (*P* < 0.01); (**B**) the expressions of Bcl-xL significantly suppressed, and the ratio of Bax/Bcl-2 significantly augmented in SHR controls compared with WKY controls (*P* < 0.01), and reversed in SHR+LA and SHR+HA (*P* < 0.01); (**C**) Survivin expression significantly down-regulated in SHR (*P* < 0.05) and reversed in SHR+HA (*P* < 0.05); (**D**) Caspase-3 expression augmented in SHR (*P* < 0.05) and suppressed in SHR+LA and SHR+HA (*P* < 0.05). Values, mean ± SEM; *n* = 3; *P < 0.01 *versus* WKY controls; #*P* < 0.01 *versus* SHR controls; δ*P* < 0.01 *versus* SHR+LA; WKY: Wistar; SHR: spontaneously hypertensive rats; LA: low dose of Aliskiren; HA: high dose of Aliskiren.

As indicated by the semiquantitative analysis of the apoptosis associated proteins in cardiomyocyte by Western blot, the ratio of p-AKT/AKT decreased by 32% in SHR controls when compared with WKYs, but was up-regulated to the normal level in SHR+LA and SHR+HA (*P* < 0.01), but with no significance between SHR+LA and SHR+HA (*P* > 0.05; Fig. 2A).

The suppressions of Bcl-xL was observed in SHR controls when compared with WKYs (*P* < 0.05), while significant enhancements were seen in SHR+HA (*P* < 0.01). The ratio of Bax to Bcl-2 was significantly up-regulated in SHR controls (*P* < 0.01) and significantly down-regulated in SHR+LA and SHR+HA when compared with SHR control or WKY (Fig. 2B).

The expression of survivin was significantly decreased by 61.2% in SHR controls (*P* < 0.01), but was up-regulated by 201.2% in SHR+HA when compared with SHR controls (*P* < 0.01; Fig. 2C).

It was also found that the expression of cleaved caspase-3 was significantly up-regulated by 94.5% in SHR control, but was down-regulated by 42.8% and 63.4% in SHR+LA and SHR+HA respectively (*P* < 0.01; Fig. 2D).

These data suggested that PI3K-AKT pathway, mitochondria-associated apoptotic signalling pathway and survivin pathway were involved in the pathophysiological changes in SHR controls, SHR+LA and SHR+HA.

### Aliskiren significantly ameliorated the enhanced autophagy in cardiomyocyte

**Fig. 3:**
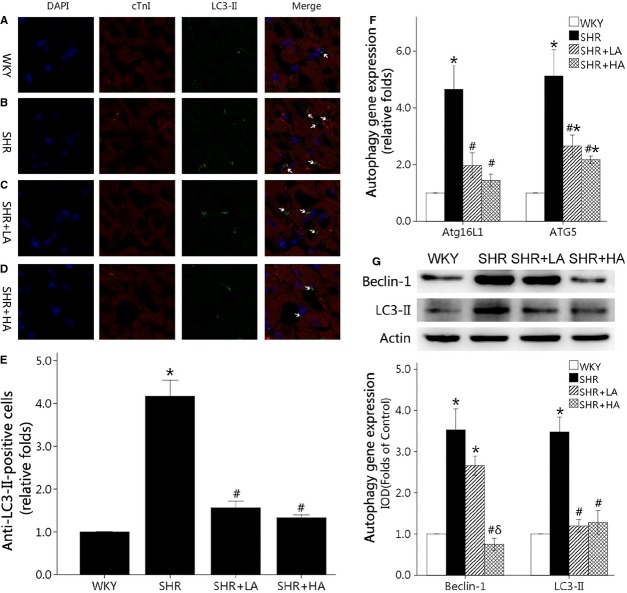
Cardiomyocyte autophagy detected by immunofluorescent labelling for LC3-II, western blot and qPCR. (**A**–**D**) LC3-II-positive aggregates in the representative immunofluorescent staining of WKY controls, SHR controls, SHR+LA and SHR+HA; (**E**) quantitative analysis of LC3-II-positive aggregates in the four groups and normalized to the WKY controls; (**F**) SHR controls showing significantly higher Atg5 and Atg16L1 expressions of mRNA than WKY ones (*P* < 0.01), and down-regulated in SHR+LA and SHR+HA (*P* < 0.01); (**G**) the expressions of Beclin-1 and LC3-II down-regulated significantly in SHR Controls (*P* < 0.01), and reversed in SHR+HA (*P* < 0.01). Values, mean ± SEM; *n* = 4; *P < 0.01 *versus* WKY controls; #*P* < 0.01 *versus* SHR controls; arrow indicating a LC3-II-positive aggregates; WKY: Wistar; SHR: spontaneously hypertensive rats; LA: low dose of Aliskiren; HA: high dose of Aliskiren.

Immunofluorescence labelling for LC3-II showed that the number of autophagy cells was nearly fourfolds in SHR controls when compared with WKYs (*P* < 0.01). SHR+LA displayed reductions in LC3-II-positive cell counts by 62.7% (*P* < 0.01), but with no significant difference between SHR+LA and SHR+HA (*P* > 0.05; Fig. 3A–E). Furhtermore, Atg5 and Atg16L1, autophagy genes, showed that nearly fivefolds in SHR controls when compared with WKYs (*P* < 0.01) and reversed to less than threefolds in SHR+LA and SHR+HA (*P* < 0.01; Fig. 3F). In additon, the expressions of Beclin-1 and LC3-II increased by 252% and 243%, respectively, in SHR controls when compared with WKYs, but were down-regulated to the normal level in SHR+HA (*P* < 0.01; Fig. 3G).

### The expression of Cx43 gap junctions in cardiomyocyte

**Fig. 4:**
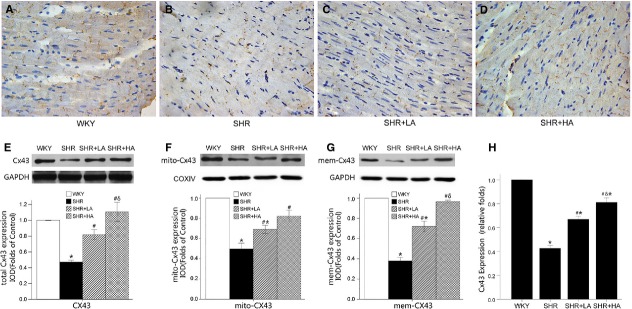
Cx43 distribution and expression in LV myocardium determined by immunohistochemistry, western blot and qPCR. (**A**–**D**) The expression and distribution of Cx43 in ventricular myocardium; (**E**–**G**) total, mitochondria and membrane Cx43 significantly down-regulated in SHR (*P* < 0.05) and reversed in SHR+LA and SHR+HA (*P* < 0.05); (**H**) SHR controls showing significantly lower Cx43 expression than WKY ones (*P* < 0.01); dose-dependent up-regulation of mRNA level in SHR+LA and SHR+HA (*P* < 0.01). Values, mean ± SEM; *n* = 3; *P < 0.05 *versus* WKY controls; #*P* < 0.05 *versus* SHR controls; δ*P* < 0.01 *versus* SHR+LA; WKY: Wistar; SHR: spontaneously hypertensive rats; LA: low dose of Aliskiren; HA: high dose of Aliskiren.

Immunohistochemistry staining of Cx43 gap junction showed the expression and distribution of Cx43 GJs in LV myocardium sections (Fig. 4A–D). To determine whether the expression of Cx43 would change among WKY, SHR controls, SHR+LA and SHR+HA, western blot and real-time RT-qPCR were conducted in all samples. It was found that the total protein levels and mRNA expressions of Cx43 decreased significantly by 53% and 57%, respectively, in SHR controls when compared with WKYs (*P* < 0.01). The total protein expressions of Cx43 increased significantly by 73% and 136% (*P* < 0.01) in SHR+LA and SHR+HA, respectively, where the mRNA levels were up-regulated by 34% and 73% (*P* < 0.01) respectively. These dose-dependent effects of Aliskiren were observed between SHR+LA and SHR+HA with significant difference (*P* < 0.01; Fig. 4E and H). To determine the protein responsible for the total Cx43 changes, the membrane Cx43 and mitochondrial Cx43 were examined separately, and a rising pattern the same as that of total Cx43 in SHR+LA and SHR+HA (Fig. 4F and G) was found.

### Aliskiren decreased the concentration of angiotensin II and the expression of AT_1_R

**Fig. 5:**
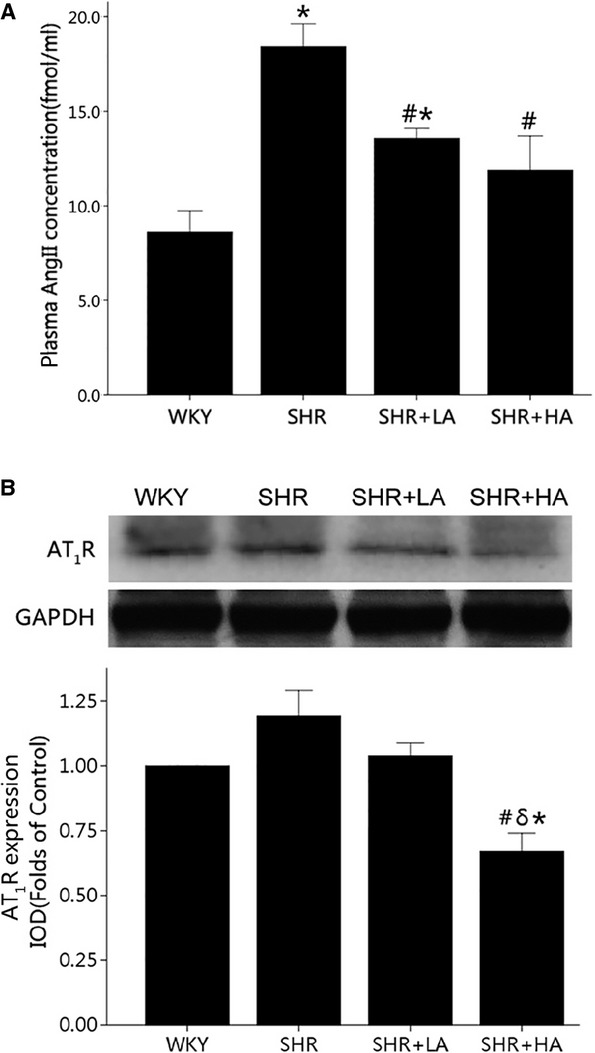
The concentration of angiotensin II and the expression of AT_1_R revealed by ELISA and western blot. (**A**) Plasma angiotensin II levels increased significantly in SHR Controls (*P* < 0.01), and reversed in SHR+LA and SHR+HA (*P* < 0.01); (**B**) the expression of AT_1_R significantly down-regulated in SHR+HA compared with SHR controls (*P* < 0.01; *P* < 0.01); values, mean ± SEM; *n* = 3; *P < 0.05 *versus* WKY controls; #*P* < 0.05 *versus* SHR controls; δ*P* < 0.01 *versus* SHR+LA; WKY: Wistar; SHR: spontaneously hypertensive rats; LA: low dose of Aliskiren; HA: high dose of Aliskiren.

Plasma angiotensin II levels increased significantly in SHR controls when compared with WKYs, and decreased in SHR+LA and SHR+HA by 26.3% and 35.4% respectively (*P* < 0.01; Fig. 5A). Furthermore, Aliskiren significantly down-regulated the expression of AT_1_R by 42.6% in SHR+HA when compared with SHRs controls (*P* < 0.01), while no significance was observed in SHR+LA (Fig. 5B).

## Discussion

Cardiac apoptosis and autophagy have been considered as having a crucial role during the transition from stable compensatory heart failure to decompensation in the end-stage hypertensive heart disease [1,3–5,20–22]. Cx43 has been reported to be altered in response to the over-expressed renin-angiotensin system (RAS) [23,24], mechanical stretch [25] and hypertrophy [26–28] and contributed to the myocardium apoptosis [10]. However, the exact role of Cx43 in cardiac apoptosis and autophagy during the end-stage hypertensive heart disease has remained unknown.

This study demonstrated that SBP and HW/BW% increased significantly in SHR controls when compared with WKYs and that there was an increase in plasma angiotensin II level, indicating the activation of circulating RAS in the aged SHR, as indicated previously [29–31]. Furthermore, we found that the apoptosis and autophagy of cardiomyocyte played an important role in the pathophysiological progress of hypertensive heart disease in aged SHR and suggested that the remodelling of Cx43 gap junction and the change in mitochondrial Cx43 may be involved in the myocardium autophagy to apoptosis.

Systolic blood pressure, plasma angiotensin II and the expression of AT_1_R decreased after Aliskiren treatment. Furthermore, myocardium autophagy and apoptosis declined significantly, along with the increased expression of membrane and mitochondrial Cx43. These findings suggested that Aliskiren was capable of attenuating myocardium apoptosis and autophagy in the aged SHRs, and the altered Cx43 may be one of the underlying mechanisms.

It is well recognized that the alterations of gap junction organization and connexin expression are consistent features of human heart disease. The recent studies also revealed that Cx43 in mitochondria contributes to mitochondrial potassium uptake [32] and plays a crucial role in the apoptosis [10]. However, the alterations of Cx43 in hypertensive heart disease have remained controversial. It was reported that Cx43 declined significantly in myocardium in both young (3-month-old) and old (12-month-old) SHRs and was elevated after supplement with omega-3 polyunsaturated fatty acids [33]. Bacharova *et al*. [28] also revealed that the declined expression of Cx43 was responsible for the early stages of ventricular hypertrophy. Nevertheless, Zhao *et al*. [27] and Fialova *et al*. [26] reported that Cx43 increased in the LV myocardium of SHRs and could be reversed by Losartan [27]. In this study, the expression of total, membrane and mitochondrial Cx43 decreased significantly in LV myocardium during the end-stage hypertensive heart disease, and the disordered pattern and distribution of Cx43 were also observed in SHRs.

One of the possible reasons for these controversial results was that SHRs are different in age. It is possible that when cardiomyocytes begin to suffer from increased after-load, cytokines, such as heparin-binding epidermal growth factor, act as an autocrine and local paracrine cardiac growth factor, thus resulting in the loss of gap junction proteins within a spatially confined microenvironment [34] and inducing the early stage of ventricular hypertrophy [28]. Cx43 up-regulation in the compensatory hypertrophy may represent an immediate adaptive response to the increased load [26,27], whereas the diminished expression and heterogeneous distribution of Cx43 in the decompensatory hypertrophy may play a maladaptive role in heart failure [35]. In addition, our findings indicated aged SHRs may have cell-to-cell interconnections damaged, mitochondria damnified and electrical coupling impaired, thereby contributing to cellular apoptosis of the LV myocardium in developing the end-stage hypertensive heart disease. Although the previous studies have revealed the association between the alteration of Cx43 and the apoptosis of the epithelial cells [36,37], our findings revealed, presumably for the first time, the relationship between Cx43 and the apoptosis of LV myocardium at the end-stage hypertensive heart disease in the aged SHRs.

Renin-angiotensin system activation has been reported to be an independent risk factor for cardiac remodelling and congestive heart failure during the development of hypertension, and play a crucial role in the alteration of the abundance and distribution of Cx43 in different pathophysiological states [23,25,38]. Iravanian *et al*. [23] and Sovari *et al*. [24] reported that RAS inhibition in mice with cardiac-specific angiotensin converting enzyme overexpression, which exhibited proclivity to ventricular tachycardia (VT) and sudden death because of the reduced Cx43, could increase the total amount of Cx43 and its phosphorylated counterpart, decrease VT inducibility and increase the survival rate, indicating that intervening RAS could attenuate the impairments of Cx43. In this study, we also observed the increased angiotensin II and the altered Cx43 in SHR control group.

It was reported that autophagy, which provides energy by degrading the impaired organelles, especially in ischaemia [39] and inflammation [40], played a crucial role in the cellular survival. However, the excessive autophagy could have harmful effect and induce cellular apoptosis. Previous studies have demonstrated that angiotensin II could induce the down-regulation of miR-30 [41], increase mitochondria oxidative stress [42], thereby causing myocardium autophagy, which indicated that RAS activation could result in the cellular autophagy. Furthermore, the cellular levels of Cx50 and Cx43 could be up-regulated by blocking the autophagy-related protein [13] and are directly dependent on the ubiquitinylation of plasma membrane connexins [14]. On the basis of the data presented in this study, we supposed that the increased angiotensin II might up-regulate myocardium autophagy, decrease the expression of Cx43 and contribute to the myocardium apoptosis in the aged SHRs.

Aliskiren, a direct renin inhibitor newly developed, was reported to have therapeutic effect on hypertension [15,16], stroke [43] and hypertrophy [17], but fail to attenuate LV remodelling in high-risk post-MI patients who had already received standard therapy including one RAS inhibitor, which suggested that dual RAS blockade with Aliskiren would not provide additional benefit to post-MI patients [44]. Previous studies [45,46] have demonstrated that Aliskiren treatment could effectively control SBP. In this study, we found that Aliskiren administered orally at 10 and 25 mg/kg/day effectively reduced SBP by 14.1% and 17.6% respectively. In Wood's [47] study, however, Aliskiren at 10 mg/kg/day failed to achieve a significant reduction in SBP. It could be that the baseline SBP variations were responsible for these divergent conclusions. In Wood's SHR, the baseline SBP was near 140 mmHg, while in Kelly's and this study, it was near 200 mmHg; it was difficult for antihypertensive medication to lower SBP to normal range. This could also be because that the treatment period differed in Kelly's (16 weeks), Wood's (2 weeks) and this study (8 weeks). In general, long-acting medications tend to produce their maximal therapeutic effects with an interval of 4–6 weeks. Therefore, the baseline SBP and course of treatment could be the reasons responsible for the significant difference.

In this study, it was considered that Aliskiren therapy not only lowered the SBP, but also down-regulated the plasma angiotensin II concentration and the expression of AT_1_R, especially in the high dose group, which might decrease the myocardium autophagy, up-regulate the expression of Cx43 and then induce a decrease in cardiomyocyte apoptosis in SHRs.

It could be concluded that Aliskiren could decrease myocardium autophagy, ameliorate Cx43 remodelling and reduce myocardium apoptosis in aged SHRs.
